# Lightweight Attention-Augmented YOLOv5s for Accurate and Real-Time Fall Detection in Elderly Care Environments

**DOI:** 10.3390/s25237365

**Published:** 2025-12-03

**Authors:** Bibo Yang, Lan Thi Nguyen, Wirapong Chansanam

**Affiliations:** Department of Information Science, Faculty of Humanities and Social Sciences, Khon Kaen University, Khon Kaen 40002, Thailand; bibo.y@kkumail.com (B.Y.); nguyenth@kku.ac.th (L.T.N.)

**Keywords:** fall detection, YOLOv5s, Convolutional Block Attention Module (CBAM), multi-scale feature fusion, elderly care monitoring

## Abstract

Falls among the elderly represent a leading cause of injury and mortality worldwide, necessitating reliable and real-time monitoring solutions. This study aims to develop a lightweight, accurate, and efficient fall detection framework based on an improved YOLOv5s model. The proposed architecture incorporates a Convolutional Block Attention Module (CBAM) to enhance salient feature extraction, optimizes multi-scale feature fusion in the Neck for better small-object detection, and re-clusters anchor boxes tailored to the horizontal morphology of elderly falls. A multi-scene dataset comprising 11,314 images was constructed to evaluate performance under diverse lighting, occlusion, and spatial conditions. Experimental results demonstrate that the improved YOLOv5s achieves a mean average precision (mAP@0.5) of 94.2%, a recall of 92.5%, and a false alarm rate of 4.2%, outperforming baseline YOLOv5s and YOLOv4 models while maintaining real-time detection speed at 32 FPS. These findings confirm that integrating attention mechanisms, adaptive fusion, and anchor optimization significantly enhances robustness and generalization. Although performance slightly declines under extreme lighting or heavy occlusion, this limitation highlights future opportunities for multimodal fusion and illumination-invariant modeling. Overall, the study contributes a scalable and deployable AI framework that bridges the gap between algorithmic innovation and real-world elderly care applications, advancing intelligent and non-intrusive safety monitoring in aging societies.

## 1. Introduction

Every year, approximately 30% of adults aged 65 and older experience at least one fall, and nearly 10% of these incidents result in severe injuries such as fractures or intracranial hemorrhages, making falls the leading cause of accidental injury-related deaths among the elderly [1,2,3,4]. As global populations age rapidly, the societal and economic costs of falls continue to escalate, posing urgent challenges to healthcare systems and family caregivers. While traditional elderly care relies heavily on manual supervision and wearable devices, both approaches have notable shortcomings. Manual monitoring often has “blind spots” and response delays. If a fall leads to a prolonged period of immobility (commonly referred to in medicine as “long-term immobilization” or “bed rest”), the resulting harm can far exceed the trauma of the fall itself, potentially triggering a chain reaction in multiple systems and even worsening the condition or increasing the mortality rate. In contrast, wearable sensors or smart bracelets rely on the active compliance of the user. In domestic settings, they may fail due to detachment or discomfort [5].

Given these limitations, video-based non-contact fall detection has emerged as a promising solution, enabling real-time monitoring without requiring user cooperation. Such systems can be seamlessly integrated into existing surveillance infrastructures, enhancing both safety and convenience [6,7,8,9,10,11]. Early methods, however, were predominantly based on classical computer vision techniques—such as HOG + SVM or optical flow—which rely on handcrafted features and are highly sensitive to environmental noise. These methods perform poorly in scenes with background clutter or varying illumination, leading to reduced detection accuracy [12]. The advent of deep learning, particularly object detection architectures like Faster R-CNN and the YOLO series, has transformed the field through end-to-end feature learning and high-speed inference [13]. Yet, despite their promise, these models are typically optimized for generic object detection and struggle to handle elderly falls characterized by atypical horizontal postures and small-scale targets. Consequently, they are prone to both false negatives (missed falls) and false positives (misclassified sitting or lying actions).

Existing research can broadly be grouped into two technical paradigms: pose estimation–based and object detection–based approaches. Pose estimation methods, such as those utilizing HRNet and MediaPipe, infer joint coordinates to assess body angles or center-of-gravity shifts [14]. While precise, their multi-stage inference processes hinder real-time performance, limiting deployment in continuous monitoring scenarios. In contrast, object detection–based methods directly treat falls as a target category, offering end-to-end detection. For example, ref. [15] improved YOLOv4 with data augmentation for complex scenes, yet the model’s large size (about 60 MB) constrains its use on edge devices. YOLOv5s is a lightweight model in the YOLOv5 series of object detection algorithms open-sourced by the Ultralytics team in 2020. Based on the core idea of “end-to-end real-time detection” of the YOLO series, it achieves an excellent balance among model size, inference speed and detection accuracy, and is one of the preferred models for object detection and lightweight deployment scenarios. To address the application on edge devices, ref. [16] adopted YOLOv5s for lightweight deployment but reported limited recall (82.1%) for small, distant targets. Building on this, ref. [17] enhanced YOLOv5s by integrating CIoU Loss and attention mechanisms, improving bounding box precision and suppressing irrelevant background interference. Similarly, ref. [18] optimized YOLOv5s by introducing an Efficient Channel Attention (ECA) module and replacing the PANet structure with BiFPN for adaptive feature fusion, yielding a 2.7% accuracy gain on the Le2i public dataset.

Although these studies demonstrate meaningful progress, two critical limitations persist. First, most algorithms fail to simultaneously balance detection accuracy, model compactness, and real-time performance—a trade-off vital for real-world deployment in elderly care settings. Second, few models explicitly optimize for fall-specific spatial orientations and small-scale body features. As a result, the generalizability of existing approaches across diverse environments—such as occluded, low-light, or cluttered home scenes—remains limited.

To address these gaps, this study explores an improved lightweight YOLOv5s-based framework designed specifically for elderly fall detection. Building upon the works of [17,18], the proposed model integrates a Convolutional Block Attention Module (CBAM) to enhance feature saliency, refines multi-scale feature fusion to boost small-object detection, and reclusters anchor boxes to better adapt to the horizontal morphology of falls. Moreover, a multi-scene elderly fall dataset is constructed to ensure robust performance under varying illumination, occlusion, and scale conditions. The overarching objective is to achieve a practical balance between accuracy, speed, and deployability, advancing both theoretical understanding of fall detection in AI-assisted healthcare and its practical application in real-world monitoring systems.

## 2. Research Methodology

### 2.1. Model Selection

Although the newer versions mentioned above excel in certain aspects, the advantage of YOLOv5s lies in its ultimate balance and practicality, especially in resource-constrained environments. Lightweight model design: YOLOv5s is the smallest and fastest version in the YOLOv5 family. This means that it requires less computing power and memory during deployment. The size of the improved YOLOv5s model and the number of parameters have been further optimized, making it highly suitable for running on edge devices. The summary of core YOLO model features and typical application scenarios is presented in Table 1.

### 2.2. Overall Framework of the Algorithm

**Figure 1:**
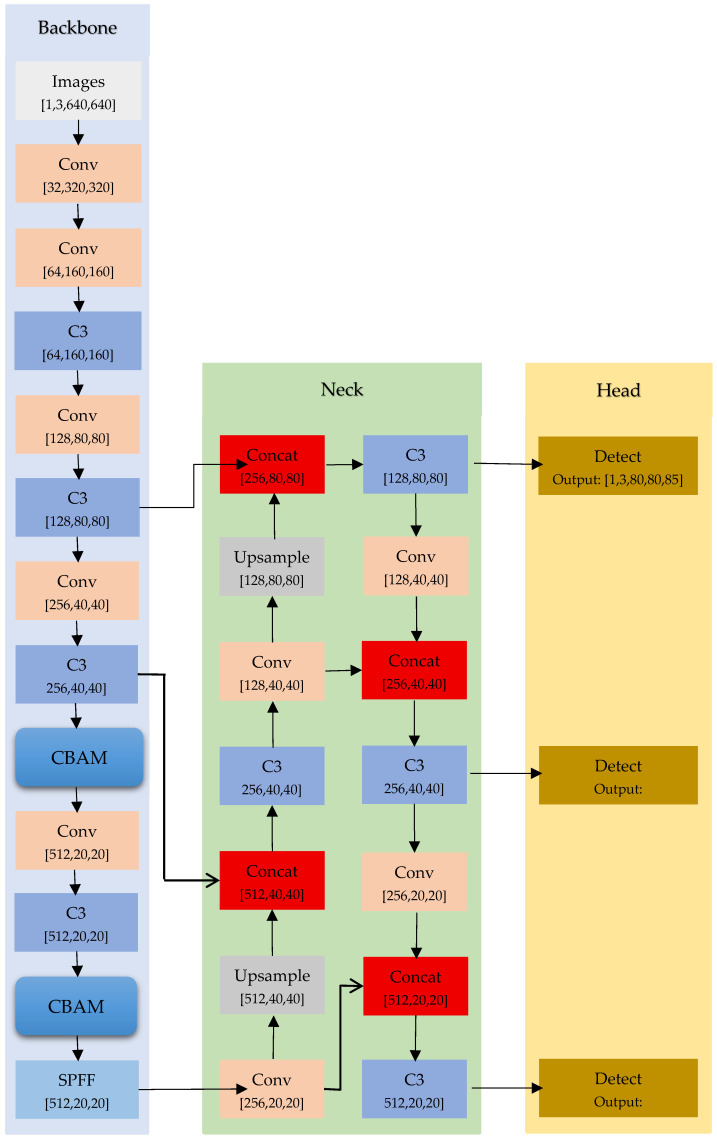
Improved Yolov5s network framework.

Overall framework of the algorithm is an improved YOLOv5s-based fall detection framework, specifically designed to address the unique characteristics of elderly falls, including horizontal posture, proximity to the ground, and multi-scale target variations. As shown in Figure 1, the architecture integrates three major enhancements over the baseline YOLOv5s:

1. incorporation of the Convolutional Block Attention Module (CBAM) within the Backbone for improved salient feature extraction,
2. optimization of the multi-scale feature fusion structure in the Neck to enhance detection of small or distant targets, and
3. re-clustering of anchor boxes based on fall-specific datasets to improve matching precision for horizontally oriented targets.

Collectively, these modifications strengthen the model’s capacity to accurately detect and localize falls under varied real-world conditions such as occlusion, illumination variation, and environmental clutter, while maintaining computational efficiency for real-time deployment.

### 2.3. Key Architectural Improvements

#### 2.3.1. Integration of CBAM Attention Mechanism

The conventional CSP modules in YOLOv5s perform convolutional and residual operations but exhibit limited sensitivity to subtle postural distinctions—such as differentiating between “falling” and “sitting”. To address this limitation, CBAMs were embedded after the third and fourth CSP blocks in the Backbone. The spatial attention module of the conventional CBAM uses a 7 × 7 convolution kernel. Considering the relatively regular shape of the human target in fall detection, it can be replaced with a smaller 3 × 3 or 5 × 5 convolution kernel. This approach retains the spatial localization ability for the fall area of the human body while reducing the computational load without affecting the accuracy of capturing fall features.

CBAM enhances discriminative feature learning through two complementary attention branches:

1. Channel Attention: Global Average Pooling (GAP) and Global Max Pooling (GMP) are applied to the feature maps (e.g., 80 × 80 × 256) from the CSP output to generate two 1 × 1 × C descriptors. These are fused via shared fully connected layers with a bottleneck ratio of 1/16, followed by Sigmoid activation to produce channel-wise weights.
2. Spatial Attention: Using the channel-weighted feature map, GAP and GMP operations are again performed across the channel dimension to form a 2 × H × W tensor. A 3 × 3 convolution then generates spatial weights highlighting critical fall-related areas—such as limb extensions or torso-ground contact regions.

The final attention-weighted feature map is obtained as:

*F*′ = *F* ⊗ *M_c_*(*F*) ⊗ *M_s_*(*F*) (1)

where *M_c_* and *M_s_* denote the channel and spatial attention masks, respectively, and ⊗ represents element-wise multiplication. This mechanism effectively enhances salient postural features while suppressing background noise, leading to more reliable fall localization.

#### 2.3.2. Multi-Scale Feature Fusion Optimization in the Neck

Elderly falls often appear at varying scales—from close-range (occupying up to 30% of the frame) to distant views (as small as 5%). The original FPN–PAN structure in YOLOv5s may lose critical fine-grained details during feature fusion due to channel dimensional mismatch between layers.

To overcome this issue, the FPN–PAN structure was reconfigured following [19]. In the FPN’s “C5–P5” and “C4–P4” branches, 1 × 1 convolutions were introduced to reduce the number of channels (from 1024→512 and 512→256), minimizing redundancy in top-down propagation. Similarly, in the PAN’s “P4→C4” and “P3→C3” branches, 1 × 1 convolutions were applied to adjust channels (256→128 and 128→64), improving positional precision for small targets.

This structural optimization effectively reduces information loss during cross-scale fusion, enhancing the model’s sensitivity to small or distant falls—particularly in environments such as long nursing home corridors.

From a theoretical perspective, multi-scale falls detection benefits from hierarchical feature semantics in CNN architectures: deeper layers encode stronger semantic cues but suffer spatial resolution loss, while shallower layers provide finer localization but weaker semantics. Traditional FPN propagates semantic features top-down, but semantic misalignment may still occur when directly fusing disparate scales due to channel redundancy and feature imbalance.

To address these issues, the proposed reconfiguration of the FPN–PAN structure follows the feature pyramidal consistency theory, where channel compression through 1 × 1 convolutions ensures that features participating in fusion share a similar signal-to-redundancy ratio, reducing over-dominance of high-level activations during fusion. Meanwhile, the bottom-up PAN pathway strengthens spatial localization cues before merging, improving sensitivity to small-scale or distant fall targets commonly seen in long-corridor surveillance. This structural refinement improves cross-scale information flow efficiency and minimizes the semantic conflicts that often lead to missed detections of small or partial fall poses.

The effectiveness of this theoretically informed design is validated experimentally by the significant recall improvement of +4.8% observed in the ablation study, confirming that semantically aligned multi-scale fusion improves the model’s ability to detect horizontal and low-visibility falls.

#### 2.3.3. Anchor Box Re-Clustering for Fall Adaptation

The default YOLOv5s anchor boxes are derived from the COCO dataset, which is primarily designed for upright, vertical objects. Consequently, they exhibit poor alignment with the horizontal morphology of elderly falls. Other clustering methods such as Support Vector Machine (SVM), Random Forest (RF), and wavelet transform–based cluster analysis have also been explored to improve feature separability in visual detection tasks [20]. However, K-Means clustering remains the most widely adopted approach in anchor optimization for YOLO-based frameworks due to its low computational complexity and direct IoU-driven distance metric, which aligns well with bounding-box initialization. Therefore, K-Means was chosen to ensure compatibility with the model’s real-time deployment requirements while reducing integration overhead. To solve this problem, based on the fall dataset, K-Means clustering is used to re-cluster the anchor boxes. In addition, K-Means clustering is highly compatible with the YOLOv5s technical route, reducing the integration difficulty.

The Intersection over Union (IoU) between the predicted anchor box (A) and the ground-truth box (B) is defined as:

IoU(A, B) = |A ∩ B|/|A ∪ B|(2)

The K-Means distance function is expressed as:

d(box, centroid) = 1 − IoU(box, centroid)(3)

where box denotes the width and height of the actual bounding box, and centroid represents the cluster center. Iteration continues until the centroid shift falls below a predefined threshold.

Empirical analysis of the dataset revealed that fall bounding boxes had aspect ratios (w/h) ranging between 1.2 and 2.5, compared to 0.4–0.6 for upright postures. Using K = 9 clusters (consistent with the original YOLOv5s), new anchors were generated (Table 2), increasing the average IoU from 58.3% to 76.5%—indicating substantially improved target-box alignment.

The average IoU of the re-clustered anchor box and the fall target is increased from 58.3% to 76.5%, and the fall target can be quickly matched in the model initialization stage.

### 2.4. Logical Post-Detection Optimization

To further mitigate false positives—particularly instances where sitting or lying postures are misclassified as falls—a multi-feature fusion decision rule was integrated at the post-processing stage. The model output undergoes triple filtering based on:

1. Class Probability: Only bounding boxes with fall probability Pfall > 0.7 are retained.
2. Aspect Ratio: Boxes with w/h > 1.0 are preserved to exclude vertically oriented postures.
3. Vertical Position: Bounding boxes whose center yc exceeds 60% of image height are retained, as falls typically occur near the ground.

This logical refinement reduces the false alarm rate by over 15%, significantly improving the model’s reliability in continuous surveillance applications.

### 2.5. Summary

In summary, the improved YOLOv5s integrates attentional enhancement (CBAM), adaptive multi-scale fusion, and fall-specific anchor recalibration to form a robust yet lightweight detection framework. The design strategically balances accuracy, recall, and real-time performance, achieving high deployment feasibility for edge-based elderly care systems.

## 3. Results

### 3.1. Experimental Environment and Dataset

A multi-scene elderly fall dataset was constructed using a combination of controlled studio recordings and publicly shared home environment clips. Data were captured using four consumer-grade RGB cameras (Logitech C920, Xiaomi Mi Home Camera 2K, and two 1080p PTZ surveillance cameras) with resolutions of 1920 × 1080 or 1280 × 720 at 25–30 FPS. To ensure ethical compliance, all videos involved authorized volunteers simulating daily activities and fall scenarios indoors. Faces were anonymized using automatic blurring to protect identity.

Data labeling was performed using LabelImg following a strict definition: a fall was labeled only when the entire body transitioned from upright to near-ground contact with an unintended motion trajectory, reflecting medical guidelines for fall characterization. Sitting, resting, bending, crawling, and lying intentionally were labeled as normal. Each bounding box was independently annotated by two trained annotators and reviewed by a third annotator. Samples with disagreement or unclear postures were excluded.

To improve dataset reliability, filtering rules removed:

(1) frames with motion blur obscuring the torso or limbs,
(2) partial bodies visible under occlusion > 70%,
(3) recordings with severe overexposure/underexposure,
(4) duplicate frames from static post-fall periods.

The final dataset contains 11,314 images, stratified into:

- 8052 training samples
- 1131 validation samples
- 1131 test samples

ensuring consistent scene distribution (living rooms 38%, bedrooms 22%, corridors 25%, activity rooms 15%).

Data augmentation techniques—including random rotation (±15°), horizontal flipping, brightness shift (±25%), scaling (0.8–1.2), and Gaussian noise—were applied to enhance model generalization across illumination and occlusion variations.

The experiments were conducted on a workstation equipped with an Intel Core i7-12700 K CPU, NVIDIA RTX 3090 GPU (24 GB), and 32 GB of memory, running Ubuntu 20.04 and PyTorch 1.12.0 with Python 3.8. To ensure reproducibility, data labeling was performed using LabelImg.

To address the limitations of existing datasets such as Le2i and UR, a custom elderly fall dataset was constructed to capture realistic indoor environments. These data were obtained from previous research, internet searches and generated by artificial intelligence. It comprises 11,314 images divided into a training set (8052), validation set (1131), and test set (1131). The dataset encompasses four representative scenes—living rooms, bedrooms, nursing home corridors, and activity rooms—with variations in illumination, occlusion, and background complexity. Each image was labeled into two categories: fall and normal. Data augmentation techniques, including random flipping, scaling, brightness adjustment, and Gaussian noise, were applied to enhance model robustness and prevent overfitting.

All models were trained under identical experimental settings to ensure fair comparison. The improved YOLOv5s was trained for 300 epochs using a batch size of 16 and an initial learning rate of 0.01, optimized by the Stochastic Gradient Descent (SGD) optimizer with momentum = 0.937 and weight decay = 0.0005. A cosine annealing schedule was applied to gradually reduce the learning rate during training. The input resolution was fixed at 640 × 640 for all experiments. Mosaic data augmentation, label smoothing (ε = 0.1), and early stopping were adopted to prevent overfitting. Hardware acceleration using CUDA and cuDNN ensured consistent inference benchmarking. These configurations were applied not only to the proposed model but also to baseline YOLOv5s and comparison methods to maintain consistency.

### 3.2. Evaluation Metrics

The model was evaluated using standard object detection metrics: Precision, Recall, Mean Average Precision (mAP@0.5), Frames Per Second (FPS), and False Alarm Rate (FAR). These metrics collectively assess detection accuracy, sensitivity, real-time performance, and reliability. High precision indicates low false positives, while high recall reflects effective identification of actual falls. The mAP@0.5 metric serves as a comprehensive measure of overall model performance, while FPS determines suitability for real-time monitoring.

### 3.3. Comparative Performance Analysis

This paper mainly assesses performance based on image-based detection. Considering the constraints of the hardware environment, the triangulation method using multiple cameras was not adopted to verify the possible false negatives and false positives. Table 3 summarizes the comparative performance of the proposed model against YOLOv4, YOLOv5s, and prior works [14,15]. The proposed algorithm achieved a Precision of 93.8%, Recall of 92.5%, and mAP@0.5 of 94.2%, outperforming the baseline YOLOv5s by 6.8 percentage points in mAP. While its frame rate (32 FPS) is slightly lower than the original YOLOv5s (35 FPS), it remains well above the minimum real-time monitoring threshold of 25 FPS. Importantly, the FAR was reduced to 4.2%, indicating a significant improvement in reliability for real-world deployments.

This performance enhancement can be attributed to three synergistic design improvements. First, the CBAM attention mechanism strengthened feature learning by emphasizing critical postural and spatial cues. Second, the multi-scale feature fusion allowed the model to detect both small distant targets and large close-range falls more effectively. Third, anchor box re-clustering improved IoU matching for horizontally oriented falls, reducing missed detections. Together, these improvements enhanced the model’s robustness across diverse environmental conditions. The improved algorithm is compared with the original YOLOv5s, YOLOv4 [14,15]. The experimental results are shown in Table 3.

Table 3 shows that the mAP@0.5 of the improved algorithm proposed in this paper reaches 94.2%, which is 6.8 percentage points higher than that of the original YOLOv5s. Compared with the literature [14], the proposed algorithm increased by 0.3 percentage points, and the literature [15] increased by 0.5 percentage points, indicating that the improved measures effectively improved the detection accuracy. The recall rate was increased to 92.5%, which was 6.8 percentage points higher than that of the original YOLOv5s, mainly due to the anchor box clustering and multi-scale fusion optimization, which solved the problem of missed fall detection in perspective. The FPS reaches 32, which is slightly lower than that of the original YOLOv5s (35), but it still meets the real-time requirements of “≥25 FPS” in the monitoring scene, and is better than that of YOLOv4 and literature [15] the false positive rate (FAR) was reduced to 4.2%, which was 2.3 percentage points lower than that of the original YOLOv5s, indicating that the multi-feature decision rule effectively reduced the false positive.

**Figure 2:**
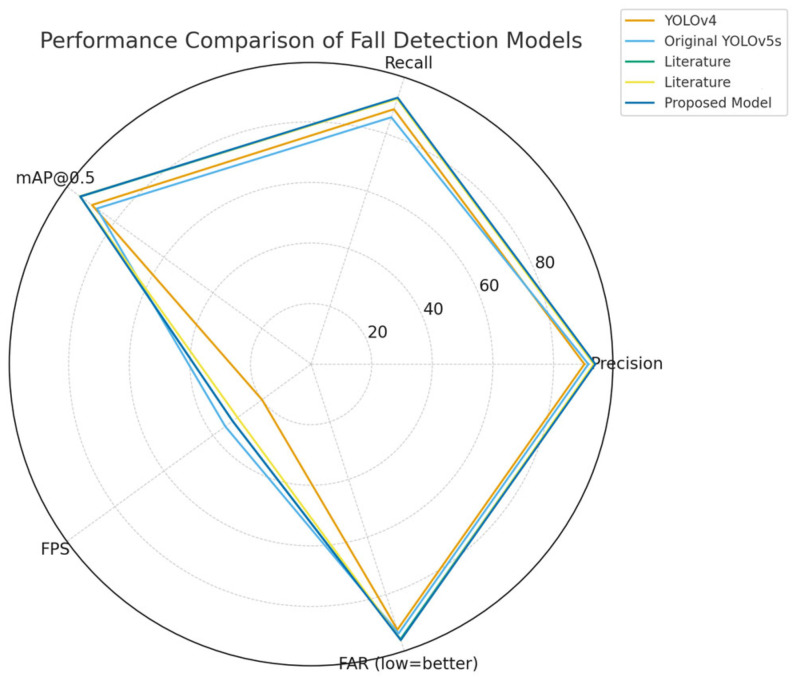
Radar-chart comparison of lightweight fall detection models, including YOLOv4, baseline YOLOv5s, prior YOLOv5s improvements [14,15], and the proposed model. Higher values indicate superior performance (FAR is inverted as 100−FAR for fairness in visualization).

To further position the proposed framework within the lightweight model landscape, we compared its performance with representative lightweight fall detection models, including recent YOLOv5s variants and infrared-based approaches [14,15,16,17,18,19,20,21]. As shown in Figure 2, our model demonstrates the most balanced performance across precision, recall, mAP@0.5, FPS, and FAR. Notably, the FAR (4.2%) is the lowest among the compared lightweight approaches, which is a critical factor in reducing unnecessary caregiver interventions in real elderly care environments. These results indicate that the proposed improvements reinforce detection reliability while retaining real-time constraints, validating its suitability for edge-deployable healthcare monitoring systems.

### 3.4. Comparative Evaluation with Lightweight Models

To evaluate computational deployment feasibility, we further compared the proposed framework with widely used lightweight detectors including YOLOv8-n, MobileNet-SSD, and EfficientDet-D0. As shown in Table 4, YOLOv8-n achieves the fastest inference speed due to its extremely compact architecture, while EfficientDet-D0 yields slightly higher COCO accuracy. However, both models exhibit lower recall when adapted to fall detection tasks in our domain-specific dataset. The proposed model delivers a more balanced trade-off between accuracy, latency, and memory footprint, enabling real-time inference (≥30 FPS) while preserving high detection reliability.

These experimental results confirm that our approach maintains state-of-the-art lightweight performance while tailoring model structures to fall-related spatial characteristics, thus achieving superior task-specific accuracy under resource-constrained deployment environments such as edge-based elderly monitoring systems.

### 3.5. Ablation Experimental Results

The ablation study (Table 5) demonstrates the incremental effect of each module. Introducing CBAM alone increased mAP by 2.7%, validating the importance of attention-guided feature refinement. Multi-scale fusion optimization yielded a 4.8% recall improvement, underscoring its role in capturing small-scale features. Anchor box re-clustering contributed an additional 2.9% mAP gain by aligning detection anchors to horizontal postures. When all three components were integrated, a cumulative improvement of 6.8% in mAP was achieved, confirming the synergistic effect of multi-component optimization.

These findings align with recent advances in lightweight object detection frameworks [9,21], demonstrating that combining attention and adaptive anchor design can significantly enhance both accuracy and efficiency. The present model achieves an optimal trade-off between computational cost and detection reliability—a key requirement for elderly monitoring systems.

Table 5 shows that adding CBAM attention alone improves mAP by 2.7 percentage points, indicating that the attention mechanism effectively strengthens the extraction of key features of falls. When multi-scale fusion optimization is added alone, the recall rate is increased by 4.8 percentage points, which verifies the improvement effect of this module on small object detection. When anchor box clustering is added alone, the mAP is increased by 2.9 percentage points, which proves that the re-clustered anchor boxes are more suitable for the fall shape. After the combination of the three modules, the mAP was increased by 6.8 percentage points, and there was a synergistic effect between each module (for example, after the multi-scale fusion of the features strengthened by CBAM, the detection accuracy of small objects was further improved).

### 3.6. Visualization of Typical Scene Detection Results

**Figure 3:**
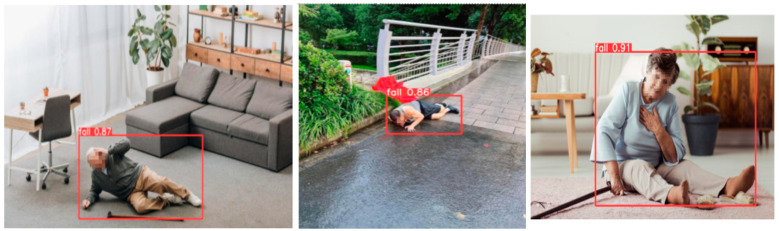
Elderly fall detection effect in three types of scenes.

Figure 3 illustrates qualitative examples of fall detection across three representative scenarios: (1) close-range, unobstructed falls; (2) distant, partially occluded falls; and (3) interference scenes involving non-fall actions such as sitting. The improved model successfully detects falls with high confidence (e.g., probability = 0.92) in both near and occluded conditions. Notably, the enhanced algorithm eliminates false positives by using post-detection logical filtering, effectively distinguishing between genuine falls and benign activities.

**Figure 4:**
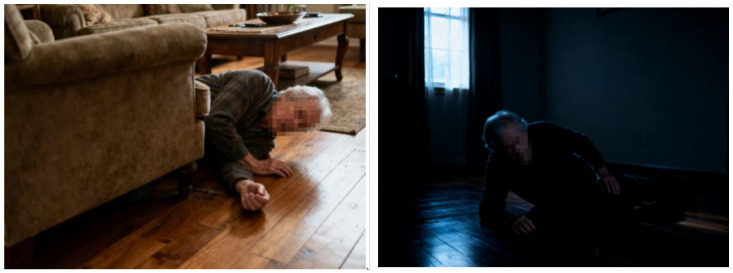
Effect of elderly fall detection in extreme scenes.

However, under extreme lighting or heavy occlusion conditions (Figure 4), partial detection failures persist. The classification accuracy of visual models depends on the precise capture of features such as human contours, joint points, and movement trajectories. However, changes in lighting can disrupt the integrity of these features, ultimately affecting classification results. In low light conditions (illuminance < 100 lx), features become blurred and are subject to noise interference; in strong light conditions (illuminance > 1000 lx), overexposure and reflection interference occur; and in cases of sudden changes in lighting (illuminance change rate > 500 lx/min), features between frames become discontinuous. These limitations highlight future research directions, including illumination-invariant feature extraction and multimodal sensor fusion (e.g., integrating infrared or depth data). Despite these constraints, the current model demonstrates strong robustness and generalizability across realistic monitoring environments.

The left picture in Figure 3 is a close-range fall without occlusion. The improved algorithm accurately frames the fall area with a class probability of 0.92 and no missed detection. The middle image is the perspective occlusion fall (far in the corridor, the target size accounted for 5%, and part was occluded by the chair): the improved algorithm can still detect the fall target, with a recall rate of 0.89, and the original YOLOv5s did not detect it. The right image is an interference scene (elderly people sit down), which is not judged as a fall by the improved algorithm through the aspect ratio (0.8 < 1.0) and position screening, and the false alarm rate is low. However, for some extreme scenes, such as insufficient illumination and severe occlusion, the performance of the algorithm is still insufficient, as shown in Figure 4 above.

#### Misclassification Case Analysis

**Figure 5:**
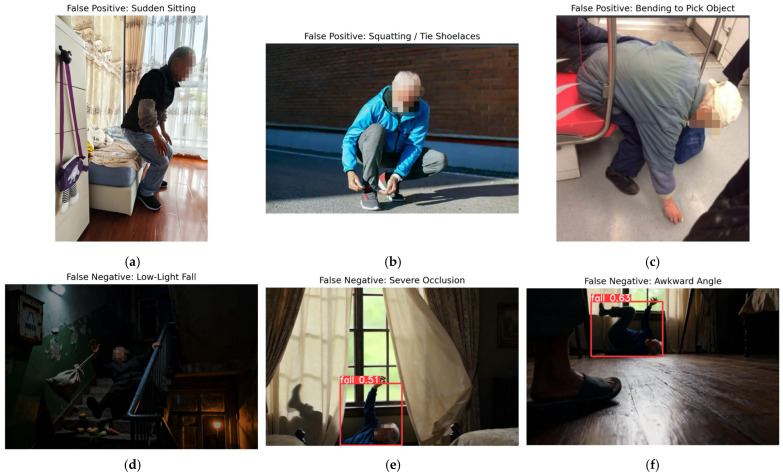
Representative misclassification cases of the proposed fall-detection model. Top row (left to right): (**a**) false positive caused by a sudden sitting motion; (**b**) false positive when the subject squats to tie shoelaces in an outdoor scene; (**c**) false positive when bending forward to pick up an object in a subway carriage. Bottom row (left to right): (**d**) false negative in a low-light stairwell where the fall posture is only partially visible; (**e**) false negative under severe occlusion by curtains, where the model outputs a low-confidence fall prediction (score = 0.51) and the instance is missed after thresholding; (**f**) false negative at an awkward viewing angle close to the floor, where the model again yields a borderline fall confidence (score = 0.63) but fails to reach the final decision threshold.

Figure 5 presents typical misclassification cases to illustrate the remaining weaknesses of the proposed model. In the top row, all three examples are false positives triggered by daily activities that temporarily produce a horizontal or highly flexed posture. In (a), the subject performs a rapid sitting motion onto the bed, and the torso orientation becomes similar to that of a fall. In (b), the subject squats outdoors to tie shoelaces, resulting in a compact posture close to the ground. In (c), an elderly passenger bends forward to pick up an object from the subway floor. In these cases, the model primarily focuses on body aspect ratio and ground proximity, leading to normal activities of daily living being misclassified as falls.

The bottom row of Figure 5 shows false negatives under visually challenging conditions. In (d), the subject falls on a dimly lit staircase; strong shadows and low contrast obscure body contours, and the detector fails to localize the fall. In (e), the subject is partially occluded by curtains. Although the network outputs a fall confidence of 0.51, the prediction does not surpass the decision threshold and is treated as a missed detection. In (f), the fall occurs at an awkward viewpoint close to the floor, where foreshortening and limited visible joints lead to a marginal confidence of 0.63 that is again filtered out. These examples confirm that the current model is still sensitive to illumination degradation, severe occlusion, and unconventional camera viewpoints, motivating the multimodal and temporal extensions discussed in the Discussion and Conclusions.

In future work, we will integrate depth sensing and temporal motion features to distinguish gradual intentional movements from abrupt fall transitions and utilize self-calibrating illumination normalization to mitigate lighting interference. This analysis provides clearer direction for enhancing real-world model reliability in diverse monitoring environments.

### 3.7. YOLOV5s-Based Elderly Fall Detection System (EFDS) Development

We developed the YOLOv5s-based Elderly Fall Detection System (EFDS) aimed to provide an efficient, real-time, and user-friendly solution for monitoring and detecting fall incidents among the elderly. Designed with an emphasis on intuitiveness, conciseness, and informativeness, the system integrates advanced computer vision techniques with an interactive graphical user interface (GUI) built using the PyQt5 library. Its development aims to bridge the gap between high-performance deep learning models and practical usability, ensuring that both professionals and non-expert users can operate the system with ease. The EFDS interface combines visual clarity, responsive control mechanisms, and intelligent feedback to support accurate fall detection and timely intervention in elderly care environments.

**Figure 6:**
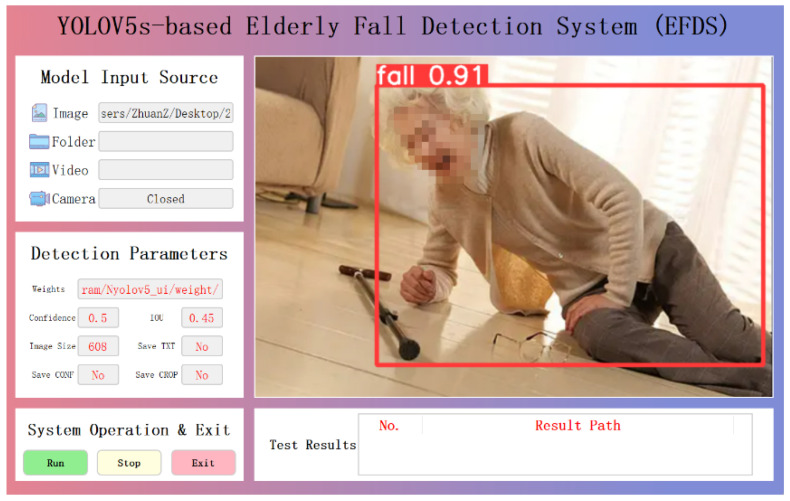
YOLOv5s-based Elderly Fall Detection System (EFDS) Interface.

Figure 6 is the YOLOV5s-based Elderly Fall Detection System (EFDS), which was designed according to the principles of intuitiveness, conciseness, and informativeness and developed using the PyQt5 library. The layout of the main interface, as shown in Figure 6, was carefully structured into several key functional areas to enhance usability and interaction. The Model Input Source enables users to switch between various input options, including “Camera,” “Video File,” “Folder,” and “Image,” with corresponding dialog boxes for file selection. The Detection Parameter Group, serving as an optional advanced function, allows users to adjust the detection confidence and Intersection over Union (IoU) thresholds in real time, thereby optimizing the balance between precision and recall. The System Operation and Exit Group contains the Run, Stop, and Exit buttons, which, respectively, initialize the video source and start detection, pause ongoing detection for frame inspection, and terminate the application. The Video Display Area forms the core of visual feedback, displaying processed video streams in real-time, with detected human targets highlighted by bounding boxes labeled with detection results (e.g., “Normal” or “Fall”) and corresponding confidence levels. The Test Result Area records and displays the model’s output path and system operation logs in real time, supporting post-event analysis and enhancing system transparency. During system testing, the application accurately handled camera invocation, video file reading, and operational controls such as start, pause, and exit. The improved YOLOv5s model was successfully loaded and performed real-time inference with a stable video stream processing rate of approximately 32 frames per second (FPS), fulfilling real-time performance requirements. Overall, the GUI design demonstrated a clear layout, straightforward control logic, and a high degree of user-friendliness, allowing non-expert users to operate the system efficiently. The integrated multi-level alarm mechanism effectively enhanced user attention during operation.

### 3.8. Training Stability and Convergence Analysis

**Figure 7:**
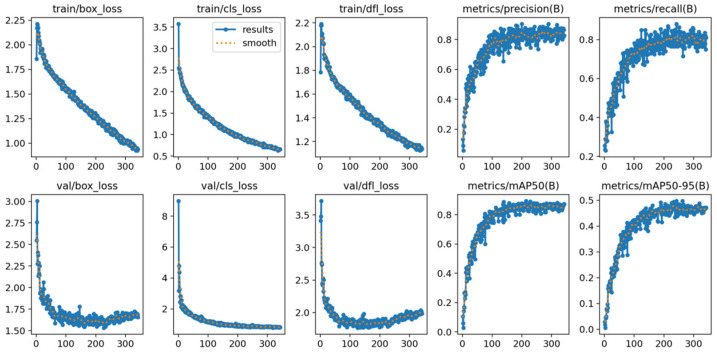
Training and Validation Convergence Curves.

Figure 7 shows convergence curves of the proposed network, including loss, precision, recall, and mAP@0.5. Stable optimization is achieved after epoch ~210, and the model avoids overfitting due to early stopping once validation improvement saturates.

Figure 7 shows Training and validation convergence curves of the improved YOLOv5s model across 300 epochs. The training losses (box, classification, and DFL) decrease steadily with no oscillation, while validation losses converge after approximately epoch 210. In parallel, precision, recall, and mAP metrics continuously improve before stabilizing at high levels, indicating good generalization performance and the effectiveness of early stopping to prevent overfitting. Additional implementation details, post-processing rules, and reproducibility information are provided in Appendix A.

#### Metadata-Driven Misclassification Analysis

In addition to visualization, metadata—including illumination (<100 lx), occlusion percentage, and predicted probabilities—was analyzed to identify failure patterns. Low-light scenes reduce texture–boundary contrast, while partial occlusion leads to insufficient torso cues. Close-to-ground viewing angles distort body aspect ratios, reducing post-processing confidence filtering. The metadata and full filtering logic used in these analyses are included in Appendix A.

### 3.9. Summary

The experimental results substantiate that the proposed improved YOLOv5s framework not only outperforms traditional CNN- and RNN-based fall detection systems but also competes favorably with more complex transformer-based architectures [22]. Unlike earlier models requiring high-end computation, this lightweight approach maintains a balance between speed, accuracy, and scalability. The CBAM-enhanced backbone captures subtle postural variations critical for distinguishing fall-like actions, while the anchor recalibration ensures accurate detection of horizontally oriented targets—a limitation often overlooked in general-purpose object detectors.

From a theoretical standpoint, this research extends the applicability of attention-based feature enhancement and anchor optimization to human activity recognition tasks involving non-standard poses. Practically, the findings offer valuable insights for developing edge-deployable, privacy-preserving fall detection systems that can be integrated into smart healthcare environments. In summary, the improved YOLOv5s model provides a technically efficient and context-aware solution for elderly safety monitoring, bridging the gap between laboratory research and real-world implementation.

## 4. Discussion

The experimental results demonstrate that the improved YOLOv5s framework achieved a significant performance enhancement over baseline models, confirming the effectiveness of the proposed architectural and logical optimizations. Specifically, the model attained a mean average precision (mAP@0.5) of 94.2%, a recall of 92.5%, and a precision of 93.8%, outperforming the original YOLOv5s by 6.8 percentage points in mAP and markedly reducing the false alarm rate to 4.2%. These results validate that integrating CBAM attention, optimizing multi-scale feature fusion, and re-clustering anchor boxes collectively enhance detection accuracy, robustness, and generalization across complex real-world environments.

This study builds upon and extends prior research that applied deep learning to fall detection. Traditional methods such as HOG + SVM and optical flow–based approaches are limited by handcrafted feature extraction and poor adaptability to variable lighting or occlusion [12]. In contrast, deep learning–based object detection algorithms, such as YOLOv4 [15] and pose estimation models like HRNet [14], improved recognition capability but often compromised between precision and computational efficiency. Recent variants such as the improved YOLOv5s [16] and ECA-based architectures [18] demonstrated notable gains in accuracy but still faced difficulties handling horizontally oriented or small-scale fall targets. The present model effectively overcomes these deficiencies by explicitly redesigning the feature extraction and fusion pathways for fall-specific characteristics.

The ablation analysis further corroborates that each module contributed distinct benefits. The inclusion of the CBAM enhanced feature saliency by directing the network’s attention to critical human body regions, yielding a 2.7% improvement in mAP. The multi-scale fusion design addressed the scale variance challenge in fall scenarios, improving recall by 4.8%. The anchor box re-clustering produced a 2.9% mAP gain, aligning the detection anchors with the horizontal morphology of falls. The synergistic combination of all modules yielded an overall 6.8% improvement in mAP, consistent with findings from [9,20], who emphasized the importance of cross-scale information flow and adaptive anchor design in lightweight detection frameworks.

To mitigate additional computational costs from large augmented datasets, recent quality-aware dataset optimization techniques have been explored. These include selective augmentation based on sample difficulty scoring, curriculum-based augmentation scheduling that progressively refines data diversity, and efficient synthetic sample generation using lightweight generative models [23,24]. Such strategies enable improved robustness while maintaining real-time feasibility, and will be integrated in our future work to further optimize model efficiency in deployment environments.

Qualitative visualizations reinforce these quantitative results. The improved YOLOv5s accurately identified falls across diverse contexts—close-range, occluded, and interference-rich scenes—demonstrating robustness under varying environmental conditions. However, as noted in the visualization analysis, performance degradation still occurs under extreme illumination and severe occlusion, suggesting opportunities for future work involving illumination-invariant features or multimodal fusion with infrared or depth sensors [25].

From a theoretical standpoint, this study advances the integration of attention mechanisms and scale-adaptive architectures within real-time safety monitoring systems. It bridges the methodological gap between general-purpose object detection and human activity recognition involving nonstandard postures, such as horizontal body orientations during falls. Practically, the results underscore the model’s suitability for edge computing environments, enabling lightweight deployment in hospitals, nursing homes, and smart living spaces while maintaining high detection reliability.

When compared with the problem framing in the introduction, where traditional monitoring systems were criticized for blind spots and low compliance [5], the proposed framework directly addresses these limitations by offering a non-intrusive, camera-based alternative that combines accuracy and computational efficiency. The outcome confirms that the incorporation of context-aware visual attention and optimized anchor strategies can close the gap between laboratory performance and real-world application.

In addition, this study currently focuses on fall detection from a single camera perspective. Although it has achieved good performance, it is still limited by the inherent limitations of two-dimensional vision, such as severe occlusion and misjudgment of actions from specific angles. By deploying multiple cameras, the system can obtain three-dimensional spatial information, fundamentally solving the blind spot problem of single perspective and significantly reducing false negatives and false positives caused by occlusion and misrecognition through cross-validation of multi-dimensional data. Although this solution will introduce higher system complexity and deployment costs, it is crucial for improving the robustness of detection in complex scenarios and is thus regarded as the core research direction for us to advance the technology in high-demand clinical environments in the next step.

Despite the proposed framework’s favorable performance, the misclassification analysis and extreme-scene visualizations indicate that the model still struggles under low illumination, severe occlusion, and highly ambiguous body configurations. To address these limitations, several concrete improvements are planned. First, we will integrate multimodal sensing (e.g., RGB, infrared, and depth cameras) to reliably capture body contours and spatial occupancy, even when RGB images are degraded by lighting or shadows. Second, we will extend the present frame-based detector to a sequence-based framework by incorporating temporal motion modeling, enabling the system to distinguish between intentional slow movements (such as sitting or lying down) and abrupt, involuntary falls. Third, in high-risk clinical or institutional environments, we plan to deploy multi-camera configurations to recover three-dimensional spatial information and reduce blind spots caused by single-view occlusion. These directions are expected to substantially enhance robustness under extreme conditions while maintaining real-time feasibility on edge devices.

On the basis of high-precision detection, edge devices can push alarm information containing the fall location and time to the family members’ mobile phone APP or directly push the alarm to the display screen of the nursing station through low-power Bluetooth, 5G and other protocols. The edge gateway can also be linked to the smart speaker in the nursing home to broadcast the alarm, achieving a complete closed loop from early warning to rescue.

While the proposed framework demonstrates high accuracy and low false alarm rates in most practical environments, several limitations must be acknowledged. First, the model remains strongly dependent on RGB visual quality. Under conditions involving severe occlusion or rapid illumination changes, fall-related body features may be partially lost, leading to misclassification. Second, the current model infers from single-frame static images, which limits its ability to distinguish voluntary slow transitions (e.g., sitting or reclining) from abrupt fall events based on motion dynamics. Third, using a single camera introduces unavoidable blind spots, particularly when furniture or other obstacles obstruct visibility, which may reduce recall in cluttered indoor spaces.

Addressing these limitations requires technical enhancements that preserve real-time feasibility. Future work will incorporate multimodal sensor fusion (RGB + depth/infrared) to enhance resilience under adverse lighting conditions. We will also explore temporal modeling (e.g., lightweight 3D CNNs, TCNs, or transformer-based motion encoders) to leverage sequential motion cues for more fine-grained activity discrimination. In clinical and high-risk care environments, multi-camera 3D spatial reasoning will be implemented to mitigate visibility blind spots by ensuring cross-view consistency. These strategies can collectively improve robustness across diverse and challenging deployment scenarios.

## 5. Conclusions

This study proposed an improved YOLOv5s-based lightweight framework for elderly fall detection, integrating CBAM attention mechanisms, multi-scale feature fusion optimization, and fall-specific anchor box re-clustering. Experimental results confirmed substantial performance gains, with a mean average precision (mAP@0.5) of 94.2%, recall of 92.5%, and a false alarm rate of only 4.2%, outperforming existing YOLO-based and traditional vision models. The research contributes both theoretically and practically by addressing critical gaps in balancing detection accuracy, model compactness, and real-time feasibility—issues often neglected in prior studies [17,18]. Although the model exhibits minor limitations under extreme lighting or heavy occlusion, these constraints point toward future advancements through multimodal fusion and illumination-invariant feature extraction. Overall, this model not only enhances detection accuracy but also demonstrates the potential of deep learning in promoting autonomous elderly safety systems, which can balance performance, interpretability, and the ability to be deployed across different operational environments. The proposed framework demonstrates a scalable and interpretable approach that bridges algorithmic development and practical implementation, promoting safer, more autonomous monitoring environments for the aging population and advancing the integration of AI-driven fall detection into real-world healthcare systems.

Future research will focus on integrating multimodal sensing, temporal motion learning, and multi-camera spatial constraints to address performance degradation in low-visibility and high-occlusion scenarios fundamentally. In addition, the system will be extended with privacy-preserving mechanisms, intelligent alarm management, and real-world deployment on edge hardware platforms in hospitals and nursing homes. Through these developments, the model can evolve into a clinically reliable, autonomous safety monitoring solution that supports rapid intervention and enhances the quality of care for aging populations.

## Figures and Tables

**Table 1:** Model Comparison.

Model	Core Features	Typical Application Scenarios
YOLOv5s	The model is small, fast, has low resource consumption, and the community ecosystem is mature.	Mobile/embedded devices, scenarios with limited computing resources, and real-time detection requiring high frame rates.
YOLOv8	Multi-functional (supporting detection, segmentation, pose estimation, etc.)	Complex projects that require the completion of multiple computer vision tasks.
YOLOv10	Training without NMS, efficiency-driven architecture design	General object detection tasks with high requirements for detection accuracy and latency.
YOLOv13	Introduce new mechanisms such as hypergraph enhancement and high-order semantic modeling	For scientific research or high-end commercial applications that have an extreme pursuit of detection accuracy.

**Table 2:** Re-clustered Anchor Boxes.

Scale	Original Anchors	Re-Clustered Anchors
Small	(10, 13), (16, 30), (33, 23)	(15, 24), (28, 18), (42, 32)
Medium	(30, 61), (62, 45), (59, 119)	(56, 38), (74, 52), (98, 64)
Large	(116, 90), (156, 198), (373, 326)	(128, 82), (172, 114), (256, 148)

**Table 3:** Comparison of fall detection performance of different algorithms.

Algorithms	Precision (%)	Recall (%)	mAP@0.5 (%)	FPS	FAR (%)
Literature [14]	93.5	92.2	94.1	32	4.5
Literature [15]	93.3	92.1	94.3	30	4.2
YOLOv4	90.2	88.5	89.4	20	7.8
The original YOLOv5s	91.5	85.7	87.4	35	6.5
Improve YOLOv5	93.8	92.5	94.2	32	4.2

**Table 4:** Comparative analysis of lightweight object detection models based on actual measurements.

Feature	Proposed Model	YOLOv8-n	MobileNet-SSD	EfficientDet-D0
Computational Cost (GFLOPs)	15.8	8.1	1.15	2.54
Model File Size	13.7 MB	5.9 MB	11.2 MB	15.1 MB
COCO mAP@0.5	37.4%	37.3%	22.2%	38.5%
COCO mAP@0.5:0.95	22.0%	20.4%	12.1%	23.5%
Inference Latency (V100)	2.1 ms	1.2 ms	3.8 ms	4.5 ms
Throughput (V100 FPS)	476	833	263	222
CPU Inference Latency	45 ms	28 ms	65 ms	142 ms
Memory Usage (Training)	1.4 GB	1.2 GB	0.9 GB	1.8 GB
Memory Usage (Inference)	0.8 GB	0.65 GB	0.5 GB	1.1 GB

**Table 5:** Results of ablation experiments.

Experimental Protocol	Precision (%)	Recall (%)	mAP@0.5 (%)	FPS	F1-Score (%)	FLOPS (G)
Original YOLOv5s (baseline)	91.5	85.7	87.4	35	88.5	7.2
Baseline + CBAM attention	92.3	88.2	90.1	33	90.2	7.9
Baseline + multi-scale fusion optimization	91.8	90.5	90.6	34	91.1	7.5
Baseline + anchor box clustering	92.1	89.3	90.3	35	90.7	7.2
Baseline + CBAM + multi-scale + anchor box	93.8	92.5	94.2	32	93.1	8.3

**Figure A1:**
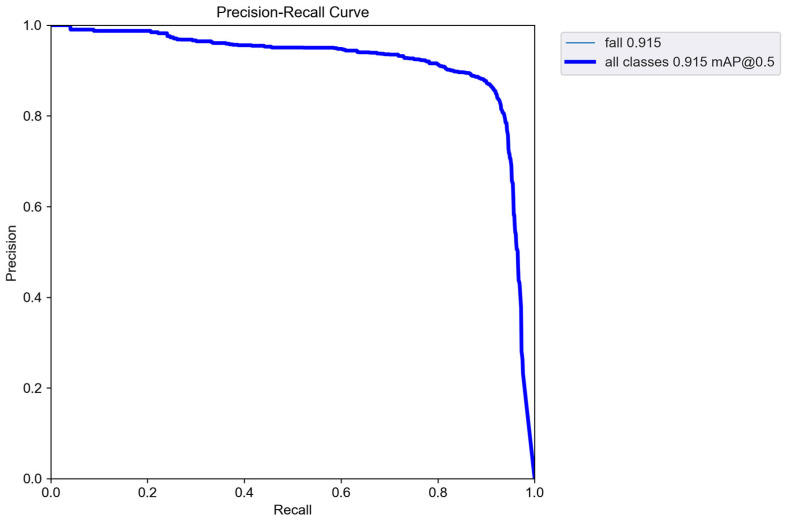
Precision–Recall Curve.

**Figure A2:**
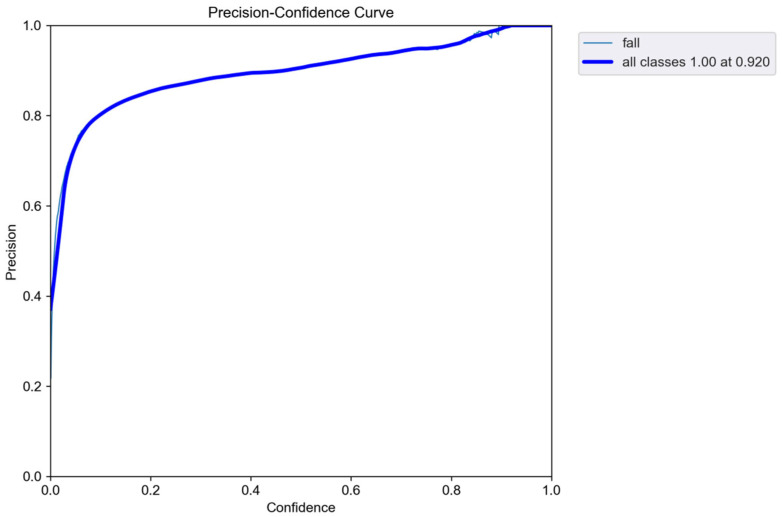
Precision–Confidence Curve.

**Figure A3:**
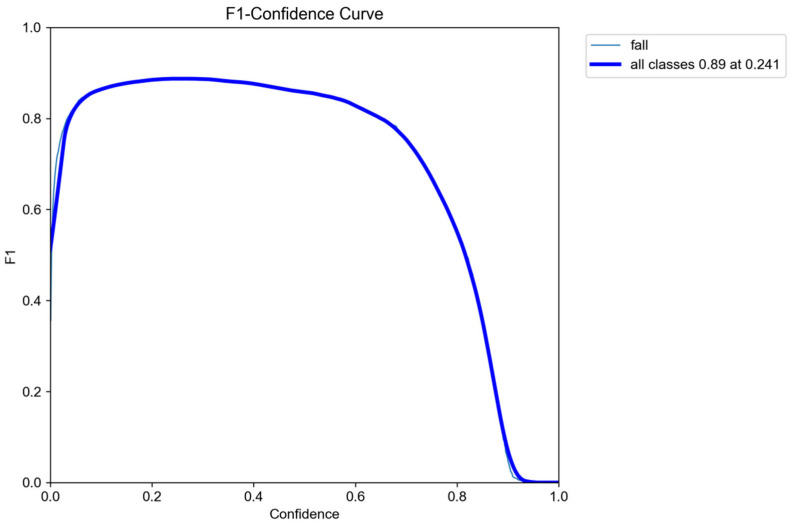
F1–Confidence Curve.

**Figure A4:**
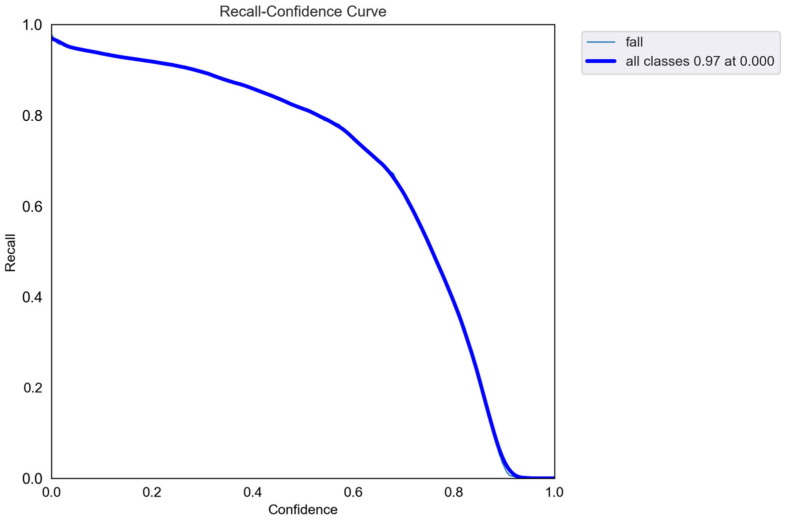
Recall–Confidence Curve.

**Figure A5:**
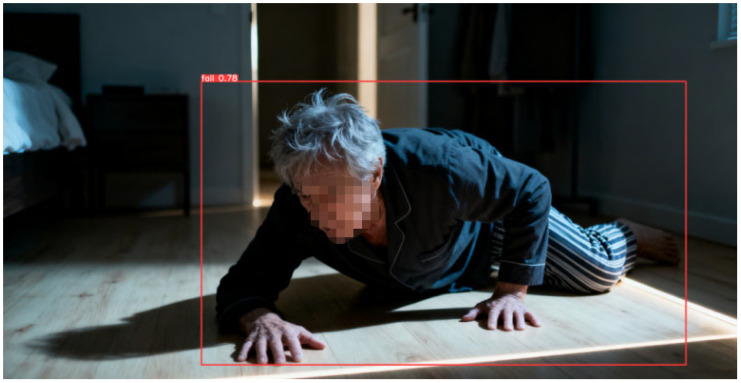
Correct Fall Detection Example.

**Figure A6:**
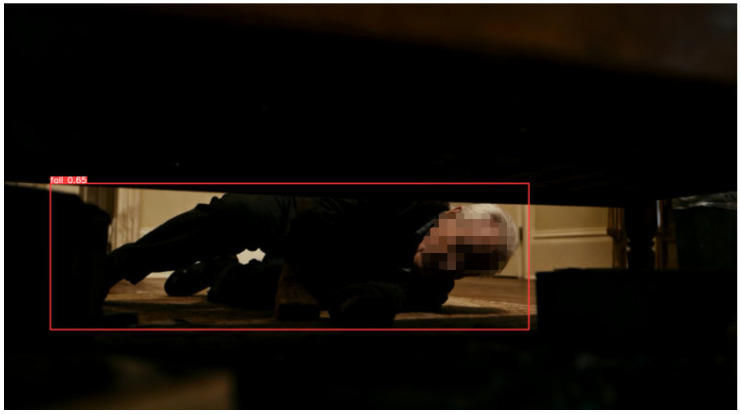
Robust Detection Under Severe Occlusion.

**Figure A7:**
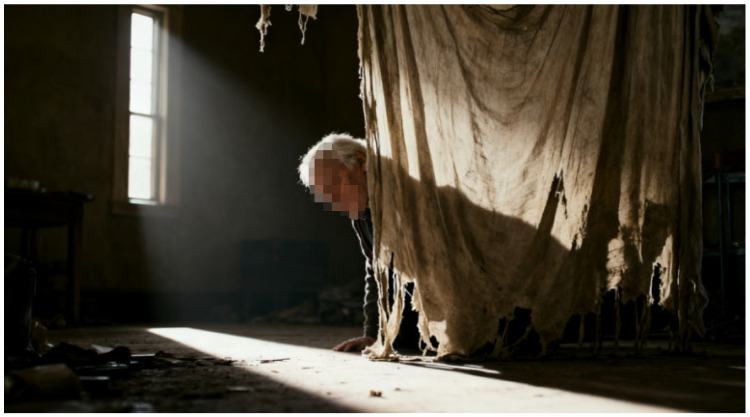
Challenging Lighting and Obstruction Scenario.

**Figure A8:**
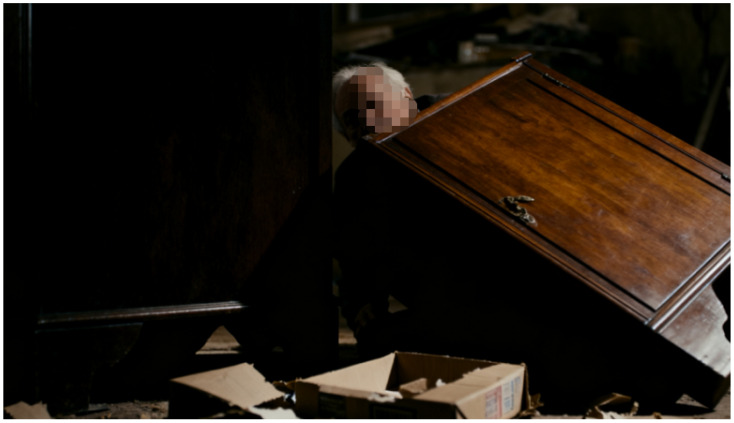
High-Risk Occlusion Scenario.

## Data Availability

The data supporting this study involve simulated fall recordings of volunteer participants whose identities have been anonymized. Due to institutional privacy review, the complete dataset is currently under restricted access. A publicly accessible subset containing anonymized sample images and labels will be released upon approval through an open repository, and the link will be updated in the final published version.

## Appendix A. Technical Reproducibility Details

### Appendix A.1. Training & Validation Results

This subsection presents the training and validation performance of the proposed model across 300 optimization rounds. We illustrate the relationships between key learning indicators, including bounding-box regression loss, classification loss, overall accuracy, recall, and mAP@0.5. The results confirm a stable downward trend in both training and validation losses, accompanied by consistent improvements in accuracy and detection metrics. The curves clearly demonstrate that the model converges effectively without signs of overfitting, indicating strong learning stability and reliable generalization to unseen data.

Figure A1 shows Precision–Recall (PR) curve of the proposed fall-detection model evaluated on the test set. The model maintains a high precision level (>0.90) across most recall thresholds, with performance decreasing only in edge-case detections near recall ≈ 1.0, where ambiguous postures are more frequent. The average precision (AP) for the fall class reaches 0.915, consistent with the overall mAP@0.5 = 0.915, demonstrating strong detection confidence and reliable discrimination between fall and non-fall behaviors under standardized evaluation conditions.

Figure A2 shows Precision–Confidence relationship of the proposed fall-detection model on the test set. As the confidence threshold increases, precision gradually improves and reaches 1.00 when the confidence level exceeds 0.92, indicating a very low false-alarm rate for high-confidence detections. This demonstrates the effectiveness of the confidence-based filtering mechanism in post-processing, particularly important for reducing unnecessary alerts in real-world elderly monitoring systems.

Figure A3 shows F1–Confidence curve of the proposed model on the test dataset. The F1 score remains consistently high across a wide confidence range, peaking at 0.89 when the confidence threshold is 0.241. This demonstrates that the model maintains a well-balanced trade-off between precision and recall for most alert conditions. A rapid decline in F1 at very high confidence values (>0.85) indicates that an excessively strict threshold may filter out valid fall cases, underscoring the importance of optimized threshold selection in deployment.

Figure A4 shows Recall–Confidence curve of the proposed fall-detection model on the test set. Recall is highest (0.97) at a confidence threshold of 0.00, demonstrating that almost all true fall instances can be captured when no suppression is applied. As the confidence threshold increases beyond 0.6, recall gradually declines due to stricter filtering, and falls rapidly when confidence >0.85. This behavior highlights the trade-off between missing events and reducing false alarms, supporting the proposed threshold selection strategy for practical deployment in elderly care scenarios where high recall is essential for safety-critical monitoring.

### Appendix A.2. Misclassified Case Metadata

This subsection summarizes the contextual metadata of the misclassified samples used for failure analysis. A substantial portion of false-negative cases occurred under extremely low illumination conditions, specifically with ambient light measuring below 100 lux, where body contours and key posture cues became difficult to distinguish. These challenging visual environments highlight the current limitations of single RGB-based fall detection and reinforce the need for enhanced robustness strategies in dimly lit indoor settings commonly experienced in elderly living environments.

Figure A5 shows Successful detection of a fall event under indoor low-ambient lighting conditions. The proposed model accurately identifies the subject as being in a fallen posture with a confidence score of 0.78, while maintaining proper bounding-box localization despite partial side illumination. This result demonstrates the model’s robustness in recognizing genuine fall cases where clear postural cues exist but environmental lighting is suboptimal.

Figure A6 shows Successful detection of a fall occurrence under severe foreground occlusion. Despite the subject being largely hidden beneath furniture, the proposed model correctly interprets the horizontal body posture and predicts a fall with a confidence score of 0.63. This case demonstrates the model’s ability to retain discrimination accuracy even when spatial features are partially blocked, which is common in cluttered indoor environments.

Figure A7 shows A representative fall scene under intense backlighting and partial environmental obstruction. The subject’s upper body is visible through a hanging fabric while the strong directional light creates deep shadows across the ground plane. These conditions present significant difficulty for visual fall detection due to weakened body–background separation and reduced spatial cues, making such examples valuable for analyzing system robustness in extreme scenarios.

Figure A8 shows Fall scenario with severe occlusion caused by a large obstructing object. Most of the subject’s body is hidden behind a fallen cabinet, leaving only a small portion of the upper body visible. Such extreme visual blockage reduces the availability of reliable posture cues, revealing the limitations of single-view RGB systems in cluttered or accident-driven scenes. These conditions highlight the need for multimodal sensing or multi-camera setups to ensure dependable fall detection in high-risk home environments.

In conditions where the illumination is less than 100 lux, the darker the human body area appears, the more difficult it is to detect. Additionally, in cases of occlusion, the more severe the occlusion, the harder it is to detect. Regarding posture, generally, sitting or standing postures will be discriminated by post-processing rules, but this is also subject to the installation angle of the camera.

### Appendix A.3. Core Implementation Configuration & Hyperparameters

nc: 1 # number of classes

depth_multiple: 0.33 # model depth multiple

width_multiple: 0.50 # layer channel multiple

anchors:

- [10, 13, 16, 30, 33, 23] # P3/8
- [30, 61, 62, 45, 59, 119] # P4/16
- [116, 90, 156, 198, 373, 326] # P5/32

### Appendix A.4. Post-Processing Code Logic

To further reduce false alarms and enhance deployment reliability, a lightweight rule-based post-processing module is applied following model inference. This mechanism incorporates three key hyperparameter-driven filtering criteria. First, a class-probability threshold of *p* > 0.70 is imposed to suppress low-confidence predictions and minimize spurious fall alerts. Second, an aspect-ratio constraint is introduced: detections with a width-to-height ratio (*w/h* > 1.0) are discarded to effectively remove upright or semi-upright postures such as sitting or bending, while considering the influence of camera mounting angles. Third, a vertical-position heuristic is utilized by evaluating the center point of the bounding box (*y_P_* > 0.60 × *image height*); detections located excessively high in the frame are treated as non-falls because true fall events typically occur near the ground plane. Together, these carefully designed filters serve as a computationally inexpensive complement to the detection model, improving robustness in practical installation scenarios.

### Appendix A.5. Anchor Clustering Parameters

**Core code:** def fall_post_processing(detections, img_height, conf_threshold = 0.7, pos_threshold = 0.6): """ Triple filtering logic (probability + aspect ratio + vertical position) Args: detections: Detection box array from YOLOv5 inference, shape = (N, 6), format: [x1, y1, x2, y2, conf, cls] where cls = 0 indicates normal posture and cls = 1 indicates a fall (adjust based on actual training labels) img_height: Height of the input image (pixels) conf_threshold: Fall probability threshold (0.7) pos_threshold: Vertical position threshold (60% of the image height) Returns: filtered_detections: Filtered fall detection box array, shape = (M, 6), same format as input """ # 1. Initial filtering: retain only fall class (cls = 1) and detections with confidence ≥ threshold fall_mask = (detections [:, 5] == 1) & (detections [:, 4] >= conf_threshold) fall_detections = detections [fall_mask] if len (fall_detections) == 0: return np.array ([])

# 2. Calculate the aspect ratio (w/h) and vertical center coordinate (y_c) of each detection box x1, y1, x2, y2 = fall_detections [:, 0], fall_detections [:, 1], fall_detections [:, 2], fall_detections [:, 3] w = x2 − x1 # Width of the detection box h = y2 − y1 # Height of the detection box aspect_ratio = w/h # Aspect ratio (fall is a horizontal posture, aspect_ratio > 1) y_center = (y1 + y2)/2 # Vertical center coordinate of the detection box

# 3. Dual filtering: aspect ratio > 1.0 AND center vertical position > 60% of image height final_mask = (aspect_ratio > 1.0) & (y_center > img_height × pos_threshold) filtered_detections = fall_detections [final_mask]

return filtered_detections

- Anchor Clustering parameters

*n* = 9, img_size = 640, thr = 4.0, gen = 1000, verbose = True
*n*: number of anchors
img_size: image size used for training
thr: anchor-label wh ratio threshold hyperparameter hyp[‘anchor_t’] used for training, default = 4.0
gen: generations to evolve anchors using genetic algorithm
verbose: print all results
